# Secondary metabolites and bioactivities of *Myrtus communis*

**DOI:** 10.4103/0974-8490.75449

**Published:** 2010

**Authors:** Mahmoud I. Nassar, El-Sayed A. Aboutabl, Rania F. Ahmed, Ezzel-Din A. El-Khrisy, Khaled M. Ibrahim, Amany A. Sleem

**Affiliations:** *Department of Chemistry of Natural Compounds, Dokki, Cairo, Egypt*; 1 *Department of Pharmacognosy, Faculty of Pharmacy, Cairo University, Cairo, Egypt*; 2 *Department of Pharmacology, National Research Centre, Dokki, Cairo, Egypt*

**Keywords:** Antihyperglycemic, anti-inflammatory, antinociceptive, LD_50_, *Myrtus communis*, Volatile oil

## Abstract

**Background:**

*Myrtus* species are characterized by the presence of phenolic acids, flavonoids, tannins, volatile oils and fatty acids. They are remedies for variety of ailments. This study therefore investigated medicinal effects of *Myrtus communis* L.

**Methods:**

Bioactivity studies of *Myrtus communis* L. leaves were carried out on volatile oil, 7% methanol and aqueous extracts and the isolated compounds myricetin 3-O-β-glucopyranoside, myricetin 3-O-∝–rhamnopyranoside and gallic acid.

**Results:**

Determination of the median lethal dose (LD_50_) revealed that the volatile oil, alcoholic and aqueous extracts were practically nontoxic and highly safe as no lethality was observed. The tested materials (volatile oil, alcoholic and aqueous extracts, myricetin 3-O-β-glucopyranoside, myricetin 3-O-∝–rhamnopyranoside and gallic acid) showed significant antihyperglycemic, anti-inflammatory and antinociceptive effects as compared with control groups and reference drugs.

**Conclusion:**

Administration of extracts of *M. communis* leaves could be safe at the dose used in this study.

## INTRODUCTION

Diabetes mellitus (DM) is a common disorder associated with increased mortality rate and can be identified as a group of metabolic diseases characterized by chronic hyperglycemia resulting from defects in insulin metabolism and impaired function in carbohydrate, lipid and protein metabolism.[1,2] Myrtaceae is a family comprising at least 133 genera in more than 3800 species. It has a wide distribution in warm climate regions of the world.[3,4]

*Myrtus* is a genus of flowering plants with approximately 16 species of evergreen shrubs or small trees reported in areas of the Middle East and Asia.[5,6] *Myrtus communis* L. known as true myrtle and in arabic as mirsin. The plant grows in countries bordering the Mediterranean area and west Asia; it grows spontaneously in Spain, France, Tunisia, Algeria and Morocco. The common myrtle has upright stem, eight or 10-feet high, its branches form a close full head, thickly covered with ovate or lanceolate evergreen leaves; it has solitary axillary white or rosy flowers, followed by black a several-seeded berry which is spherical in shape with dark red to violet in color. Myrtus species were reported as very rich in volatile oils,[7,8] phenolic acids as gallic and ellagic acids,[6] flavonoids,[6,9] fatty acids (FA),[10] tannins[11] and anthocyanin pigments.[12] The present study deals with isolation and identifications of secondary metabolites as well as antihyperglycemic, anti-inflammatory activity, antinociceptive activity and LD_50_ as prospective analysis.

## MATERIALS AND METHODS

All the instruments used found at National Research Centre, Cairo.

### General

NMR measurements were carried out using JEOL EX -500 spectroscopy; 500 MH_Z_ (^1^H NMR) and 125 MH_Z_ (^13^C NMR) and JEOL JNM-EX 270 spectroscopy; 270 MHz (^1^H NMR) and 67.5 MH_Z_ (^13^C NMR), mass spectra (±) ESI-MS: LCQ Advantage Thermo Finnigan spectrometer GLC instrument used was Agilent 6890N gas Finnigan- Mat SSQ 7000 spectrometer provided with FID (Flame Ionization Detector). EI ev 70, fused silica capillary column 30-m length, helium gas as a carrier gas; flow-rate (column head pressure 13 PS) and MS detector and UV spectra (OMM 7070E Shimadzu UV 240 spectrophotometer) were run.

### Plant materials

*M. communis* L. leaves were collected from El-Orman garden, Giza, Egypt. The plant samples were kindly identified by Mm. Tressa Labib, Taxonomist, El-Orman garden, Giza, Egypt. The collected samples were air dried, powdered and kept for chemical analysis.

### Preparation and GC/MS analysis of volatile oil

Five hundred grams of *M. communis* leaves was subjected to steam distillation for 5 hr to give yellow oil with pleasant odor and analyzed by GC/MS system equipped with willey 138 and NBS 75 library software was used capillary GC using DB- 5 column. Injection volume was 1.0 µl at 1:50 split. Ionization voltage 70 ev scans mass range 30-450, with temperature program 50°C/5 min, 50-160, 3°C/min and 160-260, 5°C/5 min. The essential oil was identified by matching their spectra with those recorded in the MS library and comparison with those of reference compounds.

### Preparation of aqueous and alcoholic extracts

The air-dried leaves of *M. communis* (200 gm) was extracted with warm distilled water for 6 hrs then filtered off to afford 50 gm of the extract, as well as the dried leaves of *M. communis* (200 gm) was extracted with 70% ethanol for five times to afford 150 gm of the extract.

Preparation and GLC analysis of unsaponifiable matter (USM) and FA.

Petroleum ether extract (15) was saponified to yield the USM fraction (5 gm) and FA fraction (3.9 gm). The FA fraction was methylated by refluxing in 50 ml absolute methanol and 1.5 ml conc. sulphuric acid for 2 hrs and analyzed by GLC. The column used was a capillary column 30-m length, 0.53 mm internal diameter, film thickness 1 µm, packed with HP-INWAX polyethylene glycol. The analysis was carried out at a programmed temperature: initial temperature 100°C (kept for 1 min), then increasing at a rate of 4°C/min and final temperature 220°C (kept for 20 min). Injector temperature was 275°C and detector temperature at 300°C, N_2_ was as carrier gas at flow-rate 30ml/min For GLC of USM fraction, the column used was a capillary column 30-m length HP-1 methyl siloxane, 530 µm internal diameter 0.26 µm film thickness. The analysis was carried out at a programmed temperature: Initial temperature 60°C for 2 min then increasing at a rate of 10°C/min till 280°C, N_2_ was used as carrier gas at flow-rate, 30 ml/min for GLC of FA.

#### Quantitative estimation of total phenolic content in M. communis leaves

The total phenolic content (TPC) of *M. communis* leaves was determined using Folin-Ciocalteu reagent.[13] In this method the reaction mixture was composed of (100 µl) of plant extracts and 500 µl of the Folin-Ciocalteu reagent and 1.5 ml of 20% sodium carbonate. The mixture was shaken thoroughly and made up to 10 ml using distilled water; the mixture was allowed to stand for 2 hrs then the absorbance was measured at 765 nm using spectrophotometer. A calibration curve of gallic acid which was dissolved in methanol, water (60: 40 v/v, 0.3% HCl). The content of TPCs of each extract was estimated by comparison with the standard curve generated from gallic acid.

#### Quantitative estimation of total flavonoids content in M. communis leaves

The total flavonoids content (TF) of each extract was determined spectrophotometerically using rutin as a reference compound.[14] One milliliter of the plant extract in methanol (10 mg/ml) was mixed with 1-ml aluminium trichloride in ethanol (20 mg/ml) and a drop of acetic acid, and then diluted with ethanol to 25 ml the intensity of the developed yellow color was measured at 415 nm. Were taken after 40 min against blank samples (1ml of plant extract and a drop of acetic acid, and then diluted to 25 ml with ethanol). The absorption of standard rutin solution (0.5 mg/ml) in ethanol was measured under the same conditions. All determinations were carried out in triplicate. The amount of flavonoids in *M. communis* (leaves and fruits) extracts in rutin equivalents (RE) was calculated by the following formula:

$$X\;=\;\left(A\;.\;m_{0}\right)/\left(A_{0}\;.\;m\right)$$

Where *X* = flavonoid content was expressed as milligrams of RE/milligrams of plant extracts.

*A* = absorbtion of plant extracts solution, *A_0_* = absorbtion of standard rutin solution, *m_0_* = weight of standard rutin solution in mg, m = weight of the plant extract in mg.

#### Isolation of phenolic compounds

The air-dried powder leaves of *M. communis* (1 kg) were extracted with 70% ethanol. The ethanolic extract was evaporated under reduced pressure to yield 150 g of a brown residue that was suspended in water 1000 ml and partitioned successively with petroleum ether, chloroform, ethyl acetate and *n*- butanol to afford 15, 20, 70 and 30 gm, respectively. The ethyl acetate extract (70 gm) was subjected to a polyamide 6S column and eluted with distilled water/methanol step gradient. The obtained fractions were inspected by paper chromatography using BAW and 15% Ac. OH as developing systems. The similar fractions were collected together and subjected to Sephadex LH-20 afforded gallic acid (80 mg), methyl gallate (10 mg), myricetin-3-O-β-glucoside (60 mg), myricetin-3-O-²-rhamnopyranoside (65 mg), quercetin-3-O-β-galactoside (6 mg), quercetin-3-O-β-glucopyranoside (4mg), quercitrin (6 mg), ellagic acid (40 mg), myricetin (15 mg) and quercetin (10 mg).

### Animals

Albino mice of 25-30 gm body weight and adult male Albino rats of Sprague Dawely Strain of 130-150 gm body weight were used in this study, obtained from the animal house colony of National Research Centre, Egypt. All animals were kept on a standard laboratory diet under the same hygienic conditions.

### Chemicals and kits

Metformin (Chemical Industries Development, Giza, A.R.E), alloxan (Sigma Co: Cairo, Egypt.) Biodiagnostic kit for assessment of blood glucose and glutathione levels, indomethacin (Epico, Egyptian Int. Pharmaceutical Industries Co., A.R.E.). Carrageenan Sigma Co. Tramadol^®^ (Sigma Co.). All other chemicals used in the experimental work were in analytical grade.

Doses of the tested materials and drugs were administered orally by gastric tube.[15]

### Pharmacological screening

*Median Lethal Dose* (LD_50_): determination of the LD_50_ of extracts and pure compounds of *M. communis* leaves was estimated where all doses were expressed in terms of extract weight/ animal weight.[16] Preliminary experiments were done to determine the minimal dose that kills all animals (LD_100_) and the maximal dose that fails to kill any animal. Several doses at equal logarithmic intervals were chosen in between these two doses, each dose was injected in a group of six animals by subcutaneous injection. The mice were observed for 24 hrs and symptoms of toxicity and mortality rates in each group were recorded and the (LD_50_) was calculated.

*Antihyperglycemic activity*: Male albino rats of the Sprague Dawely Strain (130-140 g) were injected intra-peritoneal with alloxan (150 mg/kg body weight) to induce DM.[17] Hyperglycemia was assessed after 72 hrs by measuring blood glucoseand after 2 and 4-week intervals. Animals were divided into seven groups, first group: diabetic rats that served as control, second group: rats that received 100 mg/kg of the aqueous extract, third group: rats that received 100 mg/kg of the alcoholic extract, fourth group: rats that received 100 mg/kg of the myrecetin 3-*O*-glucoside, fifth group: rats that received 100 mg/kg of the myrecetin 3-*O*-rhamnoside, sixth group: rats that received 100 mg/kg of the gallic acid, seventh group: diabetic rats that received 150 mg/kg b.wt. of metformin drug as reference drug. At the end of each study period, blood samples were collected from the retro-orbital venous plexus through the eye canthus of anesthetized rats after an overnight fast. Serum was isolated by centrifugation and the blood glucose level was measured.[18]

*Anti-inflammatory activity*: This effect was determined according to the method described by Winter *et al*.[19] Forty-eight male albino rats, weighing 130-150 gm were divided into eight groups, each of six animals, first group: rats that received 1 ml of saline serving as control, second group: rats that received 100 mg/kg of the aqueous extract, third group: rats that received 100 mg/kg of the alcoholic extract, fourth group: rats that received 0.1ml/kg of the oil, fifth group: rats that received 100 mg/kg of the myrecetin 3-*O*-glucoside, sixth group: rats that received 100 mg/kg of the myrecetin 3-*O*-rhamnoside, seventh group: rats that received 100 mg/kg of the gallic acid, eighth group: rats that received 20 mg/kg of the reference drug, indomethacin. One hour later, all the animals received a sub plantar injection of 0.1 ml of 1% carrageenan solution in saline, in the right hind paw and 0.1 ml saline in the left hind paw. Four hours after drug administration, the rats were sacrificed; both hind paws excised and weighed separately.

$$\%\;\mathrm{Edema}\;=\;\frac{Weight\;of\;right\;paw\;-\;weight\;of\;left\;paw}{weight\;of\;left\;paw}\;\times \;100$$

*Antinociceptive activity*: Animals were acclimatized to the laboratory conditions for at least 1 hr before testing and were used once during the experiment.

Acetic acid-induced writhing test:The first group received acetic acid, second, third, fourth, fifth, sixth and seventh groups received the extracts in the aforementioned doses, and 30 min later 0.6% acetic acid was injected i.p. (0.2 ml /mice). Each mice was then placed in an individual clear plastic observe chamber and 1 total no of writhes/30 min was counted for each mouse.[20]

#### Statistical analysis

The obtained data were analyzed by using the Student’s *t* test.[21]

## RESULTS AND DISCUSSION

Nineteen compounds were identified in the volatile oil of *M. communis* by using GC/MS analysis [Table 1]. 1, 8-Cineol (27.19%), α-pinene (25.53%), linalool (11.75%) represent the major constituents. Saponfication of the petroleum ether extract afforded the FA fraction, as well as unsaponfiable matter (USM) fraction. GLC analysis of FA revealed the presence of 11 compounds. The total identified saturated FAs (47.19%) was higher than that of unsaturated FAs (52.1%); palmitic and arachidic acids were the major saturated FAs (13.9% and 13.5%, respectively); oleic and linolenic acids were the major unsaturated FAs (18.4% and 11.4%, respectively). GLC of USM fraction revealed the presence of 19 compounds. The total identified compounds representing (97.6%) and involved cholesterol (17.26%), β-sitosterol (6.69%), in addition to long-chain hydrocarbons. Column chromatography of *M. communis* leaves lead to the isolation of gallic acid,[22] methyl gallate,[23] myricetin-3-*O*-β-glucoside,[24] myricetin-3-*O*-±-rhamnopyranoside,[25] quercetin-3-*O*- β-galactoside,[26,27] quercetin-3-*O*-β-glucopyranoside (4 mg),[28] quercitrin,[29] ellagic acid,[30] myricetin[31,32] and quercetin.[31] The TPC in *M. communis* leaves (mg gallic acid equivalent/gram of plant extract) in alcoholic, chloroformic and ethyl acetate extracts was 472.47 ± 3.73, 346.89 ± 7.56 and 714.33 ± 4.69, respectively, while the TF content in *M. communis* leaves (mg RE/ gm of plant extract) in alcoholic, chloroformic and ethyl acetate extracts was 281.15 ± 21.88, 44.78 ± 8. 98 and 153.62 ± 13.27, respectively.

**Table 1:** GC/MS analysis of the volatile oil from the leaves of *Myrtus communis*

Identified compounds	Relative area percentage	KI
α-Pinene	25.53	931
Limonene	1.6	1022
1,8-cineol	27.19	1029
Linalool oxide	0.5	1071
Linalool	11.75	1094
Fenchyl alcohol	0.16	1109
α-Terpineol	0.65	1169
cis-pinocarveol	0.43	1180
Nerol	0.37	1226
Mertenyl acetate	4.2	1239
Geraniol	0.66	1259
Linalyl acetate	3.39	1261
P-menth-1-enol	6.95	1289
trans - Pinocarveyl acetete	0.15	1290
α-Terpinyl acetate	1.4	1351
Neryl acetate	0.51	1398
Trans caryophyllene	0.54	1406
α-Humulene	0.63	1448
Total percentages of identified	90.24	

^*^Identification was achieved by comparison of Kovat index (KI) with those obtained from the NIST/ NBS libraries spectra and those reported by Adams.[33]

### Bioassay

Study of the acute toxicity of the volatile oil, aqueous and alcoholic extracts of *M. communis* leaves were safe and their LD_50_ were 6.4, 10 and 10 gm/kg, respectively. The antihyperglycemic activity of volatile, aqueous and alcoholic extracts, myricetin-3-*O*- glucoside, myricetin- 3-O- rhamnoside and gallic acid are presented in [Table 2] with percentage 42.7, 1.5, 54, 41.3, 35.3 and 34.4%, respectively. It could be deduced that the alcoholic extract exhibited the highest antihyperglycemic activity as compared with the control. Results of the anti-inflammatory activity of volatile oil, aqueous and alcoholic extracts, myricetin-3-*O*- glucoside, myricetin- 3-O- rhamnoside and gallic acid [Table 3] with percentage 59.4, 50.7, 56.6, 53.9, 46.8 37.4%, respectively. It could be concluded that, the volatile oil exhibited the highest anti-inflammatory activitycompared with indomethacin used as a reference drug. Antinociceptive activity of volatile oil, aqueous and alcoholic extracts, myricetin-3-*O*- glucoside, myricetin- 3-*O*- rhamnoside and gallic acid [Table 4] with percentage 50, 46.1, 51, 40.9, 35.6 and 26.7%, respectively.

**Table 2:** Antihyperglycemic activity of aqueous and alcoholic extracts, volatile oil and isolated compounds of *Myrtus communis* leaves on blood glucose level in male albino rats (n=10)

Time Compounds for diabetic rats	Zero		2 weeks		4 weeks	
	% of change	M ± S.E	% of change	M ± S.E	% of change	M ± S.E
Control	-	82.4 ± 2.3	-	84.1 ± 2.7	-	83.6 ± 2.1
Diabetic non treated rats	-	248.3 ± 6.8	-	256.4 ± 5.9	-	261.8 ± 7.8
Aqueous extract (100 mg/kg)	-	251.9 ± 5.6	2.1	201.3 ± 7.9*	1.5	163.4 ± 6.1*
Alcoholic extract (100 mg/kg)	-	262.7 ± 8.4	30.6	182.4 ± 6.2*	54.0	121.2 ± 5.1*
Volatile oil (0.1 ml/kg)	-	267.1 ± 8.9	28.4	191.2 ± 6.7*	42.7	153.1 ± 5.9*
Myricetin-3-0-glucoside (20 mg/kg)	-	244.9 ± 8.8	26.8	179.2 ± 4.2*	41.3	143.7 ± 4.8*
Myricetin-3-0- rhamnoside(20 mg/kg)	-	266.2 ± 8.4	16.7	221.7 ± 6.8*	35.3	172.3 ± 5.4*
Gallic acid(20 mg/kg)	-	249.1 ± 8.2	20.7	197.6 ± 7.3*	34.4	163.5 ± 6.1*
Metformine (150 mg/kg)	-	259.2 ± 9.6	37.8	161.3 ± 8.4*	65.4	89.7 ± 2.3*

t: translocation; inv: inversion.

* Statistically significant from zero time at *P* < 0.01; Percentage of change calculated with reference to the control group (healthy rats that received 1 ml saline and kept under the same hygienic conditions)

**Table 3:** Acute anti-inflammatory activity of aqueous, alcoholic extracts, volatile oil and certain compounds isolated from *Myrtus communis* leaves in comparison with indomethacin in male albino rats (n=6)

Group	Dose in mg/ kg.b.wt.	% edema	
		Mean + S.E.	% of Change
Control	1 ml saline	59.4 ± 1.5	-
Aqueous extract	100	29.3 ± 0.5*	50. 7
Alcoholic extract	100	25.8 ± 0.6*	56. 6
Volatile oil	0.1 ml	24.1 ± 0.7*	59.4
Myricetin 3-0- glucoside	20	27.4 ± 0.3	53.9
Myricetin 3-0- rhamnoside	20	31.6 ± 0.9	46.8
Gallic acid	20	37.2 ± 1.3	37.4
Indomethacin	20	21.2 ± 0.4*	64.3

* Statistically significant at *P* < 0.01; Percentage of change calculated with reference to the control group (healthy rats that received 1 ml saline and kept under the same hygienic conditions)

**Table 4:** Antinociceptive activity of aqueous and alcoholic extracts, volatile oil and isolated compounds of *Myrtus communis* leaves on number of abdominal constrictions in mice (n=6)

Animal group	Dose (mg/kg)	No. of abdominal constrictions	% inhibition
Control	1	48.6 ± 1.3	-
Aqueous extract	100	26.2 ± 0.3	46.1
Alcoholic extract	100	23.8 ± 0.5	51.0
Volatile oil	0.1 ml	24.3 ± 0.4	50
Myricetin 3-0- glucoside	20	28.7 ± 0.7	40.9
Myricetin 3-0- rhamnoside	20	31.3 ± 0.9	35.6
Gallic acid	20	35.6 ± 1.1	26.7
Tramadol	20	18.7 ± 0.4	61.5

Percentage of inhibition calculated with reference to the control group (healthy rats that received 1 ml saline and kept under the same hygienic conditions)
